# N-acetylcysteine protects against star fruit-induced acute kidney injury

**DOI:** 10.1080/0886022X.2016.1256315

**Published:** 2016-11-15

**Authors:** Maria Heloisa Massola Shimizu, Pedro Henrique França Gois, Rildo Aparecido Volpini, Daniele Canale, Weverton Machado Luchi, Leila Froeder, Ita Pfeferman Heilberg, Antonio Carlos Seguro

**Affiliations:** a Department of Nephrology, School of Medicine, University of São Paulo, São Paulo, Brazil;; b Nephrology Division, Federal University of São Paulo, São Paulo, Brazil

**Keywords:** Acute kidney injury, acute oxalate nephropathy, N-acetylcysteine, oxidative stress, star fruit

## Abstract

**Background:** Star fruit (SF) is a popular fruit, commonly cultivated in many tropical countries, that contains large amount of oxalate. Acute oxalate nephropathy and direct renal tubular damage through release of free radicals are the main mechanisms involved in SF-induced acute kidney injury (AKI). The aim of this study was to evaluate the protective effect of N-acetylcysteine (NAC) on SF-induced nephrotoxicity due to its potent antioxidant effect.

**Materials and methods:** Male Wistar rats received SF juice (4 mL/100 g body weight) by gavage after a 12 h fasting and water deprivation. Fasting and water deprivation continued for 6 h thereafter to warrant juice absorption. Thereafter, animals were allocated to three experimental groups: SF (*n* = 6): received tap water; SF + NAC (*n* = 6): received NAC (4.8 g/L) in drinking water for 48 h after gavage; and Sham (*n* = 6): no interventions. After 48 h, inulin clearance studies were performed to determine glomerular filtration rate. In a second series of experiment, rats were housed in metabolic cages for additional assessments.

**Results:** SF rats showed markedly reduced inulin clearance associated with hyperoxaluria, renal tubular damage, increased oxidative stress and inflammation. NAC treatment ameliorated all these alterations. Under polarized light microscopy, SF rats exhibited intense calcium oxalate birefringence crystals deposition, dilation of renal tubules and tubular epithelial degeneration, which were attenuate by NAC therapy.

**Conclusions:** Our data show that therapeutic NAC attenuates renal dysfunction in a model of acute oxalate nephropathy following SF ingestion by reducing oxidative stress, oxaluria, and inflammation. This might represent a novel indication of NAC for the treatment of SF-induced AKI.

## Introduction

Star fruit (SF) (Averrhoa carambola) is an attractive and popular fruit originated from Asia and cultivated throughout many tropical and subtropical zones. It is a member of the *Oxalidaceae* family and can be classified into two categories: the sour type—richly flavored, commonly prepared as juice and with more oxalic acid; and the sweet type—mild flavored, usually consumed as fresh fruit and with less oxalic acid.^1^

Since the neurotoxic effect of SF was first reported in 1980,^2^ several case-reports and case-series have been published showing mild to severe neurotoxicity in patients with chronic kidney disease stage 3–5 (CKD) after ingestion of fresh fruit or juice.^3–5^ Intoxication from SF may lead to a variety of neurological symptoms, including hiccups, vomiting, insomnia, asthenia, mental confusion, seizures, coma, and death.^4^ Incoercible hiccups are the most common symptom of neurotoxicity and usually do not respond to medications.^6^ Seizures and coma usually heralds fatal outcomes.^6^^,^^7^ The mortality rate after SF intoxication is high and ranges from 20% to 40%.^7^

In the past 15 years, SF nephrotoxicity has been described in several patients with normal renal function.^8^^,^^9^ The large amount of oxalate present in SF products associated to the demonstration of diffuse calcium oxalate deposition in renal tubules suggest that acute oxalate nephropathy is involved in SF-induced acute kidney injury (AKI).^9^^,^^10^ Moreover, oxalate might also induce direct renal tubular damage through the release of free radicals leading to necrosis and apoptosis.^11–13^

N-acetylcysteine (NAC) is a pharmaceutical drug and nutritional supplement largely used as a mucolytic agent, in the management of paracetamol (acetaminophen) overdose and in the prevention of radiocontrast-induced nephropathy. NAC is an antioxidant thiol that can enter to the chain of glutathione synthesis and serves as a source of sulfhydryl groups for the cells, acting as a scavenger of reactive oxygen species (ROS).^14^ The aim of this study was to evaluate the protective effect of NAC on SF-induced nephrotoxicity due to its ability to decrease oxidative stress generated in the setting of acute oxalate nephropathy.

## Material and methods

### Animals

The study was performed using adult male Wistar rats weighing between 230 and 400 g, obtained from the animal facilities of the University of São Paulo School of Medicine. Animals were housed under standard laboratory conditions, kept on a 12 h light/dark schedules and provided with food and water *ad libitum*. Room temperature was maintained at 23 °C. The study protocol was reviewed and approved by the local institutional ethics committee.

### Experimental protocol

After a 12 h overnight fasting and water deprivation, star fruit juice was administered via a metal oral-gastric tube at a dose of 4 mL/100 g body weight (BW). Fast and water deprivation were continued for another 6 h to warrant juice absorption. Thereafter, rats were allocated to three experimental groups: SF (*n* = 6), fed with tap water; SF + NAC group (*n* = 6), treated with NAC (4.8 g/L) in drinking water; and Sham (*n* = 6): subjected to no interventions. After 48 h, inulin clearance studies were performed to determine renal function (glomerular filtration rate—GFR).

Rats were fed with SF juice after a 12-h overnight fasting and water deprivation, because as reported in previous animal studies and case reports, oxalate-induced nephropathy might need large amounts of oxalate ingested associated with fasting and dehydration state, the latter conditions enhancing oxalate absorption.^8–10^

**Figure 1.**
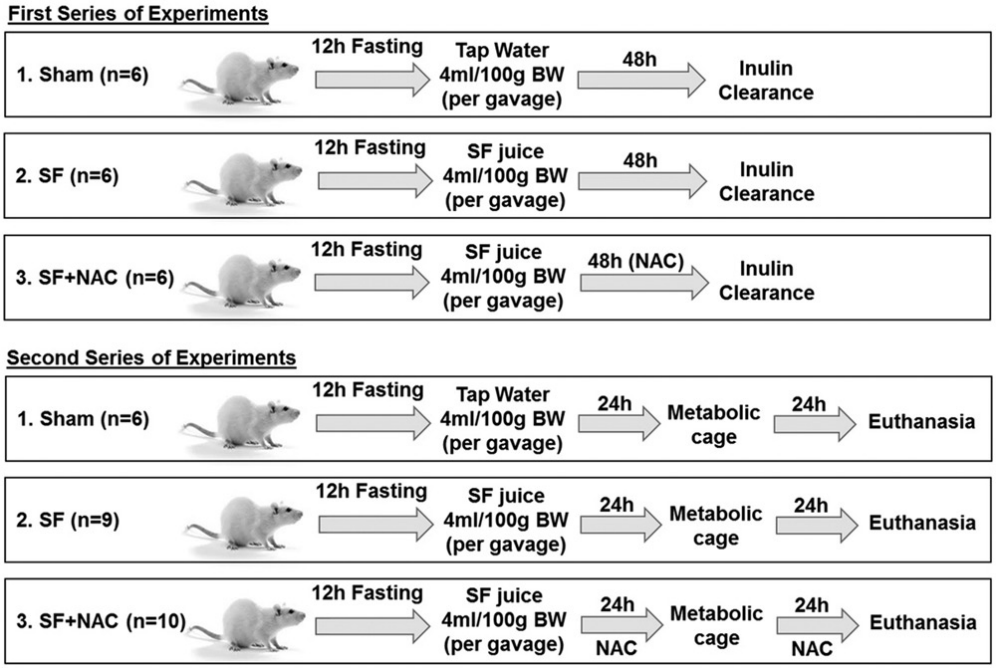
Experimental protocol: Wistar rats received Star fruit (SF) juice (4 mL/100 g body weight-BW) by gavage after a 12 h fasting and water deprivation. Animals were allocated to three groups: 1. SF; 2. SF + N-acetylcysteine (NAC): received NAC (4.8 g/L) in drinking water for 48 h after gavage; 3. Sham: control group. Two series of experiments were conducted for inulin clearance and metabolic cage studies, respectively.

In a second series of experiment, 24 h after SF administration, nine rats from SF group, 10 rats from SF + NAC group and six Sham rats were individually housed in metabolic cages for a 24 h-period in order to assess daily water ingestion, daily urinary volume, total urinary oxalate excretion, serum thiobarbituric acid reactive substances (TBARS), and serum reduced glutathione (GSH). Serum TBARS and GSH were evaluated at 24 and 48 h after SF administration, using blood samples obtained from tail vein. Figure 1 illustrates the experimental protocol.

### Inulin clearance studies

On the day of the experiment, animals were anesthetized intraperitoneally with sodium thiopental (50 mg/kg BW). Trachea was cannulated with a PE-240 catheter, and spontaneous breathing was maintained. To control mean arterial pressure and allow blood sampling, a PE-60 catheter was inserted into the right carotid artery. For the infusion of inulin and fluids, another PE-60 catheter was inserted into the left jugular vein. In order to collect urine samples, a suprapubic incision was made, and the urinary bladder was cannulated with a PE-240 catheter. After the surgical procedure was completed, a loading dose of inulin (100 mg/kg BW diluted in 0.9% saline) was administered through the jugular vein. Subsequently, a constant infusion of inulin (10 mg/kg BW in 0.9% saline) was started and was continued at 0.04 mL/minute throughout the experiment. Three urine and two blood samples were obtained during the experiment. Blood and urine inulin were determined using the anthrone method. GFR are expressed as mL/min/100 g BW.

At the end of the inulin clearance studies, the kidneys were perfused with PBS solution (0.15 M NaCl and 0.01 M sodium phosphate buffer, pH 7.4). For histological examination, right kidney was dissected out, cleaned of connective tissue and a fragment was fixed in 10% neutral-buffered formalin solution. The kidney block was dehydrated in graded alcohol, embedded in paraffin, and cut at 4 μm sections.

### Evaluation of biochemical parameters and reactive oxygen metabolites

Plasma and urinary sodium and potassium were measured by flame photometry (CELM, model FC280, São Paulo, Brazil). The fractional excretion of sodium (FENa) and potassium (FEK) were calculated using the following formula:

$$\mathrm{FE}\;\mathrm{Na}\;\mathrm{or}\;K=\frac{U/P\;\mathrm{Na}\;\mathrm{or}\;K}{U/P\;\mathrm{Creatinine}}\times 100,$$

where U: urine; P: plasma

Serum levels of TBARS, which are markers of lipid peroxidation, were determined using the thiobarbituric acid assay. In brief, a 0.2 mL serum or urinary sample was diluted in 0.8 mL of distilled water. Immediately, 1 mL of 17.5% trichloroacetic acid was added. Following the addition of 1 mL of 0.6% thiobarbituric acid, pH 2, the sample was placed in a boiling water bath for 15 min, after which it was allowed to cool. Subsequently, 1 mL of 70% trichloroacetic acid was added, and the mixture was incubated for 20 min. The sample was then centrifuged for 15 min at 2000 rpm. The optical density of the supernatant was read at 534 nm against a reagent blank using a spectrophotometer. The quantity of TBARS was calculated using a molar extinction coefficient of 1.56 × 105 M^−1 ^cm ^−1^.

Reduced GSH, the major endogenous antioxidant in cells, was determined in total blood by the method of Sedlak and Lindsay.^15^ Whole blood was processed by the addition of four volumes of ice-cold 5% metaphosphoric acid and centrifuged at 4000 rpm for 10 min at 4 °C. This assay consists of the reaction of supernatants of total blood samples with Ellman’s reagent to produce a yellow pigment measured spectrophotometrically at 412 nm. Serum GSH was quantified by means of the standard curve and reported as μmol/mL. Oxidative stress index was analyzed by serum TBARS/GSH ratio.^16^

### Urinary oxalate

Urinary oxalate was measured by and enzymatic method using a kit provided by Trinity Biotech (St Louis, MO).

### Light microscopy studies

Four micromiters histological sections of renal tissue were stained with hematoxylin-eosin and examined under light microscopy and polarized light microscopy. For the evaluation of renal damage, 40–60 grid fields (×400 magnification) measuring 0.245 mm^2^ were evaluated by graded scores according to the following criteria: (0), less than 5% of the field showing tubular epithelial swelling, vacuolar degeneration, necrosis, and desquamation; (I), 5–25% of the field presenting renal lesions; (II), involvement of 25–50% with renal damage; (III), 50–75% of damaged area; and (IV), more that 75% of the grid field presenting renal lesions. The morphometric examination was blinded to minimize observer bias, i.e., the observer was unaware of the treatment group from which the tissue originated. The mean score for each rat and the mean score for each group were calculated.

### Glomerulosclerotic index

Hematoxylin-eosin stained sections were evaluated using a light microscope (×400 magnification). One hundred glomeruli per section were randomly selected and assessed by an investigator, who was blind to treatment groups. The degree of glomerular damage was evaluated according to a semi-quantitative scoring method: grade 0, normal glomeruli; grade 1, sclerotic area up to 25% (minimal sclerosis); grade 2, sclerotic area 25–50% (moderate sclerosis); grade 3, sclerotic area 50–75% (moderate-severe sclerosis); grade 4, sclerotic area 75–100% (severe sclerosis). The glomerulosclerotic index (GSI) was calculated as follows: GSI = (1 × n1)  + (2 × n2) + (3 × n3) + (4 × n4)/n0 + n1 + n2 + n3 + n4, where n*x* is the number of glomeruli in each grade of glomerulosclerosis.^17^

### Immunohistochemistry

We subjected 4-μ kidney tissue sections to immunohistochemical reaction using monoclonal antibody anti-CD68 (1:100 for 60 min at 20 °C) (Serotec, Oxford, England). Reaction products were detected by an avidin–biotin-peroxidase complex (Vector Laboratories, Burlingame, CA), and the color reaction was developed with 3,3-diaminobenzidine (Sigma-Aldrich, St. Louis, MO) in the presence of hydrogen peroxide. The sections were counterstained with the Harris hematoxylin. Using a computer coupled to a microscope (Axioskop 40; Carl Zeiss; ×400 magnification) and a digital camera, we analyzed 30 renal cortex fields and 100 glomeruli, respectively, for assessments of interstitial and glomerular macrophage infiltration. The results of the immunoreactions were quantified by counting the number of positive cells per 0.087 mm^2^ field or per glomerulus and averaging the number of cells respectively per field or per glomerulus.

### Calcium oxalate deposition in kidney samples

To evaluate the calcium oxalate deposition throughout the kidney samples, volume ratios of positive areas of renal tissue (%), determined by color difference of deposited crystals, were obtained by image analysis with the program Image-Pro Plus, version 4.1 (Media Cybernetics, Silver Spring, MD) on a computer coupled to a polarized light microscope and a digital camera.

### Statistical analysis

All quantitative data were expressed as mean ± SEM. Differences among the means of multiple parameters were analyzed by one-way analysis of variance followed by the Student–Newman–Keuls test. Values of *p* < .05 were considered statistically significant. Data were analyzed using GraphPad Prism software 5.0 (La Jolla, CA).

## Results

### Renal function evaluation

**Figure 2.**
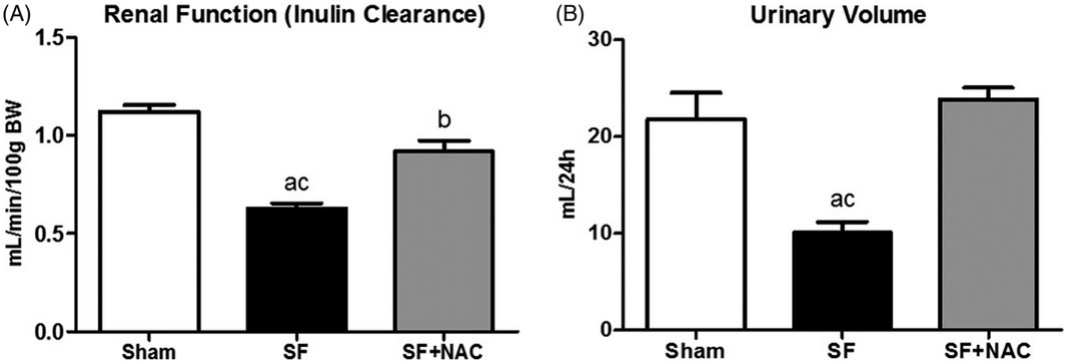
N-acetylcysteine (NAC) attenuates renal dysfunction and improves urinary volume. (A) Inulin clearance studies. (B) Urinary volume (mL/24 h). SF (Star fruit group), SF + NAC: star fruit + NAC therapy, Sham: control group. Data are mean ± SEM; ^a^*p* < .001, ^b^*p* < .01 vs. Sham; ^c^*p* < .001, ^d^*p* < .01 vs. SF + NAC.

To evaluate the efficacy of NAC therapy on SF-induced AKI, GFR was assessed by inulin clearance studies. Animals from the SF group showed markedly lower inulin clearance compared to the Sham rats. In contrast, SF + NAC group attenuated renal function deterioration following SF juice administration (Figure 2(A)). There was a significant increase in daily urinary volume after treatment with NAC compared to SF and Sham rats (Figure 2(B)). Daily water consumption was not different between groups (SF: 28 ± 2 versus SF + NAC: 30 ± 1 and Sham: 31 ± 1 mL/24 h; *p* = .4). No abnormal movements were observed after receiving SF juice. No rat from both groups died before being sacrificed after inulin experiments.

### Oxidative stress assessments

**Figure 3.**
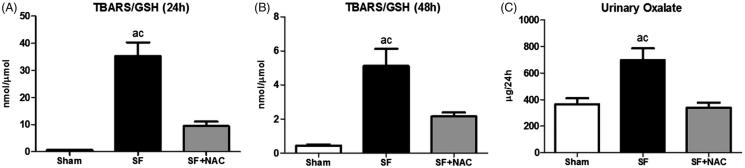
N-acetylcysteine (NAC) scavenges free radicals and improves redox balance. Plasma TBARS/GSH ratio at 24 h (A) and 48 h (B) after SF juice administration. SF (Star fruit group), SF + NAC: star fruit + NAC therapy, Sham: control group. Data are mean ± SEM; ^a^*p* < .001, ^b^*p* < .01 vs. Sham; ^c^*p* < .001, ^d^*p* < .01 vs. SF + NAC.

To determine whether the potent antioxidant effect of NAC could be the main factor involved in renal protection in the current experimental model, we assessed the serum TBARS (a byproduct of lipid peroxidation) and reduced GSH (a major endogenous antioxidant). Animals from the SF group exhibited significant increase in the plasma TBARS levels at 24 h after SF administration compared to Sham animals. NAC-treatment significantly reduced plasma TBARS levels, suggesting an improvement in the systemic oxidative stress conferred by NAC following SF-induced AKI. We found similar results, when TBARS was evaluated 48 h after SF administration. Accordingly, serum reduced GSH was lower in the SF rats compared to SF + NAC and Sham groups at 24 h and 48 h after SF juice administration. Table 1 summarizes the results of TBARS and GSH. Moreover, we have performed the plasma TBARS/GSH ratio in order to evaluate the redox balance. At both time points evaluated, there was a redox imbalance in the SF group, which was normalized by NAC treatment (Figure 3(A,B)).

**Table 1.** Biochemical and oxidative stress assessments.

Evaluated Data	Sham	SF	SF + NAC
Plasma Sodium (mEq/L)	143 ± 1	130 ± 2^a^^,^^c^	145 ± 3
Plasma Potassium (mEq/L)	3.9 ± 0.1	3.1 ± 0.1^b^^,^^c^	4.2 ± 0.3
FENa (%)	0.2 ± 0.1	1.6 ± 0.4^b^^,^^d^	1.2 ± 0.1
FEK (%)	17.5 ± 2.8	47.2 ± 3.2^a^^,^^c^	28.2 ± 2.3^b^
UK/UNa Ratio	0.71 ± 0.04	1.33 ± 0.11^b^^,^^e^	0.84 ± 0.15
TBARS (24h) (nmol/mL)	1.6 ± 0.2	21.8 ± 3.1^a^^,^^c^	6.6 ± 1.3
GSH (24h) (μmol/mL)	2.4 ± 0.1	0.6 ± 0.1^a^	0.8 ± 0.1^a^
TBARS (48h) (nmol/mL)	1.2 ± 0.2	7.4 ± 0.4^a^^,^^d^	6.0 ± 0.5^a^
GSH (48h) (μmol/mL)	2.7 ± 0.2	1.9 ± 0.4^b^^,^^e^	2.8 ± 0.1

Sham (*n* = 6): control group; SF (*n* = 9): rats who received star fruit juice (star fruit group); SF + NAC (*n* = 10): rats treated with N-acetylcysteine (NAC) after receiving star fruit juice; FENa: fractional excretion of sodium; FEK: fractional excretion of potassium; UK/UNa: urinary potassium/sodium ratio; TBARS: thiobarbituric acid reactive substances; GSH: serum reduced glutathione; Data are mean ± SEM.

a *p* < .001.

b *p* < .01 vs. Sham.

c *p* < .001.

d *p* < .01.

e *p* < .05 vs. SF + NAC.

### Renal physiology studies

Animals from the SF group exhibited lower plasma sodium and potassium as compared with the SF + NAC and Sham groups. Fractional excretion of sodium (FENa) was higher in the animals that received SF juice compared to Sham. In addition, fractional excretion of potassium (FEK) was higher in the animals from the SF group compared to Sham rats. NAC-treatment reduced the potassium excretion in this animal model. We also assessed the urinary potassium/sodium ratio (UK/Na), a simple way to estimate potassium secretion in the distal tubules. According to the previous results, UK/Na was significantly higher in the SF group compared to Sham and SF + NAC groups. Table 1 summarizes the main biochemical parameters evaluated.

SF rats exhibit higher urinary oxalate excretion, which was normalized by NAC treatment, as illustrated in Figure 3(C). Sham animals showed urinary oxalate levels of 367 ± 44 μg/24 h; *p* < .001 versus SF group and *p* = NS versus SF + NAC.

### Renal histopathology and immunohistochemistry

**Figure 4.**
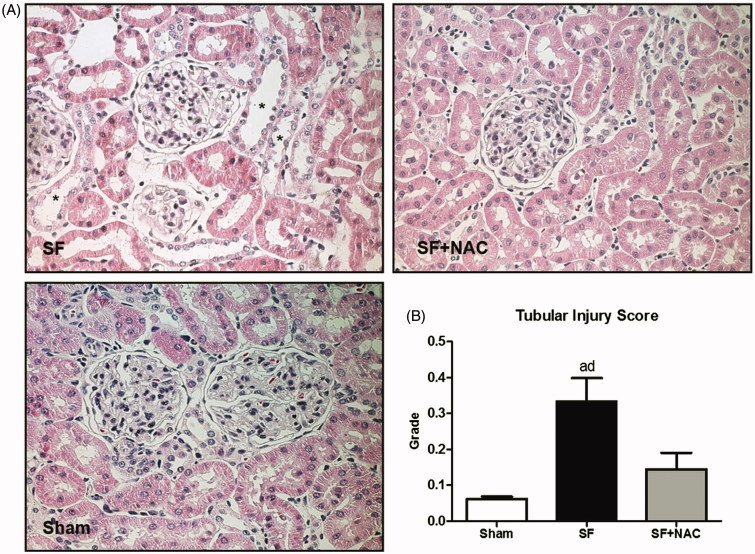
N-acetylcysteine (NAC) ameliorates renal histological lesion. (A) Histopathology of renal cortex: representative photomicrographs of kidney tissue samples. Magnification, ×400. (B) Bar graph of tubular injury scores. SF (Star fruit group), SF + NAC: star fruit + NAC therapy, Sham: control group. Data are mean ± SEM; ^a^*p* < .001, ^b^*p* < .01 vs. Sham; ^c^*p* < .001, ^d^*p* < .01 vs. SF + NAC. *flattening and dilatation of renal tubule.

**Figure 5.**
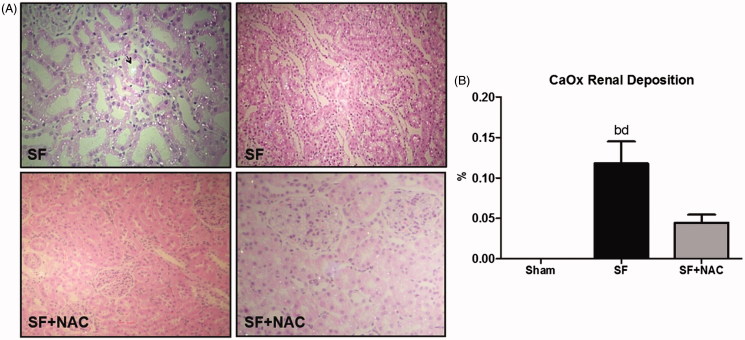
N-acetylcysteine (NAC) attenuates calcium oxalate (CaOx) deposition in renal parenchyma. (A) Representative rat kidney sections performed after star fruit juice ingestion and 48 h of water or *n*-acetylcysteine interventions. SF group exhibits intense calcium oxalate birefringence crystals deposition under polarized light microscopy and dilatation of renal tubules and tubular epithelium degeneration and flattening. Arrow: intratubular oxalate crystals. SF + NAC group shows discrete deposits of calcium oxalate and absence of tubular alterations, compatible with the lowest oxaluria observed in this group (Polarized hematoxylin and eosin; original magnification ×100 and ×400). (B) CaOx deposition throughout the kidney samples. Data were expressed in volume ratios of positive areas of renal tissue (%). SF (Star fruit group), SF + NAC: star fruit + NAC therapy, Sham: control group. Data are mean ± SEM; ^a^*p* < .001, ^b^*p* < .01 vs. Sham; ^c^*p* < .001, ^d^*p* < .01 vs. SF + NAC.

Renal histology showed acute tubular necrosis with tubular epithelium degeneration and flattening and intense dilatation of renal tubules in the SF group (Figure 4(A)). Tubular damage was not seen in SF + NAC and Sham rats (Figure 4(A)). Histopathological findings support the differences encountered in the inulin clearance studies. Renal tubular injury score was higher in the SF group and was normalized by NAC treatment (Figure 4(B)). Calcium oxalate (CaOx) crystals were absent in kidney samples of control rats. CaOx deposition was higher in renal parenchyma of animals from the SF group, which was clearly attenuated by NAC treatment. Representative kidney sections illustrate the intense deposition of CaOx birefringence crystals under polarized light microscopy in the SF group (Figure 5(A–E)).

**Figure 6.**
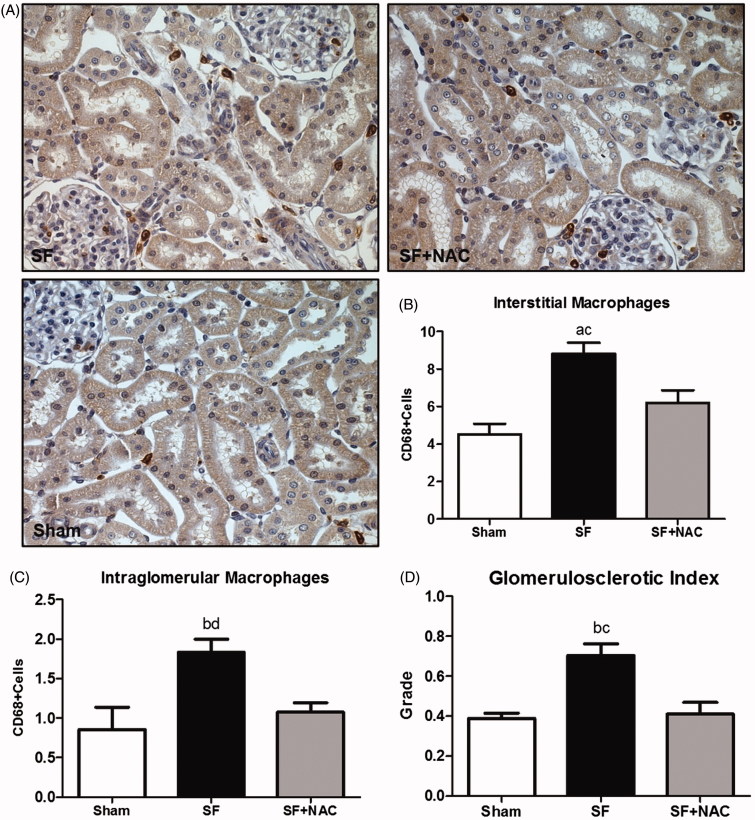
N-acetylcysteine (NAC) ameliorates renal inflammation. (A) Immunostaining (brown) for CD68 + cells in kidney cortex samples (Magnification, ×400). (B) Number of CD68 + positive cells per field (0.087 mm^2^) located in the interstitial space. (C) Number of CD68 + positive cells per glomeruli. (D) glomerulosclerotic index. SF: Star fruit group, SF + NAC: star fruit + NAC therapy, Sham: control group. Data are mean ± SEM; ^a^*p* < .001, ^b^*p* < .01 vs. Sham; ^c^*p* < .01, ^d^*p* < .05 vs. SF + NAC.

We have further evaluated the glomerular morphology using a glomerulosclerotic index (GSI). SF animals exhibited a discrete overall increase in the degree of glomerulosclerosis compared to other groups (Figure 6(D)). Moreover, there was an increased renal cortex infiltration of CD68^+ ^(macrophages) cells in the SF group compared to Sham. Macrophage infiltration was significantly lower in the NAC-treated rats (Figure 6(A,B)). Similarly, glomerular infiltration of macrophages was slightly higher in the SF group compared to Sham. NAC treatment also reduced glomerular macrophage infiltration (Figure 6(C)).

## Discussion

Since the first cases of SF nephrotoxicity have been reported,^9^ Fang et al. developed a novel rat model to address the association between SF ingestion and renal dysfunction.^10^ They showed that humans and rats seem to share similar pathological features of this syndrome.^9^^,^^18^ The mechanisms of SF toxicity to renal tubules may be multifactorial, however, renal tubular obstruction and damage induced by oxalate crystals may play a key role mediating AKI.^13^^,^^19^ Accordingly, we found that rats who received SF juice developed renal dysfunction associated with hyperoxaluria and increased oxidative stress. In the present study, NAC treatment attenuated all these alterations. To our knowledge, this is the first demonstration of NAC as a treatment for SF-induced AKI.

In the present study, the rationale of using 4 mL/100 g BW of SF juice was based on a previous experimental model of oxalate nephropathy described by Fang et al.^10^, who had reported that lower amounts of SF juice did not increase serum creatinine levels. As for NAC (4.8 g/L of drinking water), the current dose has been successfully tested by our groups in animal models of sepsis and contrast-induced nephropathy.^20^^,^^21^

Traditionally, acute oxalate nephropathy has been described secondary to substantial vitamin C ingestion, ethylene glycol intoxication and methoxyflurane anesthesia.^22–25^ However, SF consumption should also be enlisted as a potential cause of acute oxalate nephropathy, especially fresh juices made of sour SF, which contains large amount of oxalate (up to 820 mg of oxalate/100 mL of fruit preparation).^9^*In vitro* exposure of epithelial cells to oxalate leads to a dose-dependent severity of apoptosis.^18^ Moreover, oxalate induces lipid peroxidation of renal tubular membranes, generating ROS and impairing redox balance.^26^^,^^27^ Therefore, we found higher lipid peroxidation and lower antioxidant levels in the SF group after 24 h and 48 h of SF administration. Taken together, our present data provide evidence that SF, particularly due to its oxalate content, significantly increased systemic oxidative stress, which was efficiently scavenged by NAC therapy.

Several studies have validate the FENa as a pivotal diagnostic tool in the AKI setting.^28^ A FENa greater than 1% usually indicates intrinsic renal injury.^29^ In the present study, we found a mean FENa of 1.6% in the rats from the SF group, supporting the data of higher tubular injury score found in this group. Hyponatremia evidenced in the SF group was probably dilutional, considering the lower urinary volume with maintained water consumption observed in the SF group. Furthermore, we found hypokalemia associated with higher FEK and higher UK/Na ratio in rats who received SF juice, which could be the result of an increased potassium secretion in the distal nephron. In a previous study from our group, we have used the UK/Na ratio as a simple tool to estimate the enhanced delivery of sodium to the distal nephron, which is commonly a result of a functional impairment of the proximal reabsorption of sodium.^30^ Therefore, we hypothesized that oxalate toxicity affects mainly proximal tubular cells, enhancing the sodium delivery to the distal nephron.

Regarding the protective effect of NAC reducing oxaluria, we found similar results compared with the study performed by Bijarnia et al.,^26^ who showed lower oxaluria in rats treated with NAC following intraperitonial administration of sodium oxalate. In rats, as well as in humans, oxalate renal elimination kinetics comprises glomerular filtration, tubular secretion and tubular reabsorption.^31^ In mammalian kidneys, the apical membrane transporter SLC26A6 has been related to the oxalate-dependent tubular Cl^−^ reabsorption with consequent tubular oxalate secretion along the nephron (from proximal to distal tubules).^32^^,^^33^ SF animals showed more pronounced damage in the proximal tubules, which might result in higher Cl^−^ delivery to the distal nephron and, consequently, increased chloride-oxalate exchange. In NAC-treated rats, more intact proximal tubules might be associated with lower Cl^−^ delivery to the distal nephron, thus contributing to the lower oxaluria in the current experimental model. In addition, it has been demonstrated that damaged renal tubular cells can lead to tubular “back leak”,^34^ which may allow filtrated and reabsorbed oxalate back into the tubular lumen. Thus, we assume that NAC treatment might also have reduced renal tubular oxalate leakage by protecting tubular cells from oxidative stress-induced damage. However, further research needs to be undertaken to confirm this association.

We also showed a profound deposition of oxalate crystals in dilated renal tubules in the SF group. Thus, the lower urinary output encountered in the SF group might be due to the reduction in GFR associated with renal tubules obstruction by oxalate crystals. We believe that NAC treatment ameliorated AKI associated with CaOx crystals deposition and, consequently, renal tubular and glomerular injury. We showed more intense renal tubular lesion compared to the glomerular damage. In summary, we hypothesize that the reduction in GFR found in our study was mainly secondary to: (1) tubular obstruction due to CaOx crystals; (2) tubuloglomerular feedback, in which the higher distal tubular sodium chloride delivery leads to a reduction in GFR through a macula densa signaling; and (3) glomerulosclerosis.

Umekawa et al. have recently reported increased expression of monocyte chemoattractant protein-1 (MCP-1) following exposure of renal cells to CaOx-containing medium.^35^ Monocytes and macrophages recruitment have been linked to inflammation and immune reaction in several pathological conditions, including crystal-associated nephropathy.^36^ Furthermore, oxidative stress has been shown to play a major role in CaOx toxicity and chemokine stimulation.^35^^,^^37^ Therefore, the results of our research are in accordance with the abovementioned studies, whereas acute oxalate nephropathy following SF administration was associated with increased renal inflammation, which was attenuated by anti-oxidant therapy with NAC.

Previous study using an animal model of acute oxalate nephropathy reported that NAC administration reduced calcium oxalate crystals in the urine.^26^ Hence, the same group of investigators demonstrated that NAC may also reverse hyperoxaluric manifestations in rat liver.^38^ In our study, NAC treatment appeared to reduce not only oxaluria but also oxalate crystal formation in the renal tubules. One limitation of our study is that we did not determine serum or plasma oxalate.

However, plasma oxalate levels occur at concentrations 100-fold less than urine, so detection itself can be difficult and a huge variation on the reported normal range of plasma has been described.^39^ In addition, increased sample oxalate (oxalalogenesis) from the breakdown of glyoxalate or ascorbate may occur if samples are frozen and stored as well binding to plasma proteins leading to underestimation of its values. It could be speculated that animals might have experienced a peak of oxalemia after receiving SF juice, ultimately resulting in oxalate crystals deposition in the renal tubules and glomeruli. This peak of serum oxalate concentration could be followed by an increase in oxalate renal clearance as long as GFR was still preserved, normalizing serum levels. Hatch et al. observed elevations in both urine and plasma oxalate in a model of chronic hyperoxaluria with hyperoxalemia induced in rats by ethylene glycol and in a model of acute hyperoxaluria induced by intraperitoneal injection of sodium oxalate.^40^ As renal function was reduced only in the latter, it seems that the acute intraperitoneal load might have imposed a higher damage to renal function than a load derived gradually from systemic metabolism of ethylene glycol, according to the authors. Another confounding factor that must be taken into account is the net colon secretion of oxalate, which has a role in oxalate clearance when renal function is impaired.^40^ However, it is still unclear how renal and colonic excretory pathways are integrated.

In conclusion, our data show that NAC therapy attenuates renal dysfunction in a model of acute oxalate nephropathy following SF ingestion by reducing oxidative stress, decreasing oxaluria and inflammation. The present findings warrant further investigation regarding the beneficial effects of NAC in acute oxalate nephropathy or other hyperoxaluric conditions, given it is a low-cost and high safety profile medication.
